# Behçet’s Uveitis: Current Diagnostic and Therapeutic Approach

**DOI:** 10.4274/tjo.galenos.2019.60308

**Published:** 2020-06-27

**Authors:** Pınar Çakar Özdal

**Affiliations:** 1 University of Health Sciences Turkey, Ulucanlar Eye Training and Research Hospital, Clinic of Ophthalmology, Ankara, Turkey

**Keywords:** Behçet’s uveitis, imaging, treatment, biologics, prognosis

## Abstract

Behçet’s disease is a chronic, multisystem inflammatory disorder characterized by relapsing inflammation. Although its etiopathogenesis has not yet been clarified, both the adaptive and innate immune systems, genetic predisposition, and environmental factors have all been implicated. It is more frequent and more severe in males in the third and fourth decades of life. The eye is the most frequently involved organ in the course of the disease. Ocular involvement (Behçet’s uveitis) is characterized by bilateral recurrent non-granulomatous panuveitis and occlusive retinal vasculitis. Recurrent inflammatory episodes in the posterior segment may lead to permanent vision loss due to irreversible retinal damage and complications such as macular scarring, macular atrophy, and optic atrophy. Early and aggressive immunomodulatory treatment and the use of biologic agents when needed are crucial for preventing recurrences and improving visual prognosis.

## Introduction

First identified by Turkish Dermatology Professor Hulusi Behçet in 1937, Behçet’s disease (BD) is a chronic, multisystemic vasculitis of unknown etiology that involves various organs and tissues and is characterized by inflammatory episodes.^1,2^ The skin, eyes, gastrointestinal tract, and central nervous system are among the affected organs, tissues, and systems. Ocular involvement is the most common vital organ involvement and has poor prognosis, potentially culminating in blindness despite many advances in diagnosis and treatment.

### Epidemiology and Demographic Features

The disease is more common in the Mediterranean region and in Far East and Middle East countries. This geographical region falls between the 30° and 45° northern latitudes, a region that also includes the historic “Silk Road” trade route connecting the East and West and the highest HLA-B51 antigen distribution.^3,4^ The country with the highest incidence of BD worldwide is Turkey.^4^ The highest reported prevalence is in İstanbul, at 420/100,000 population.^5^ It is much less prevalent in Europe and the United States.^4,6^ Even along the Mediterranean coasts of Europe, where BD is more common compared to Northern Europe, it is much rarer than in Turkey, with a reported prevalence of 2.4-7.5/100,000.^6^

BD mostly affects the younger population between the ages of 25 and 35 years.^1,4,7^ The incidence in childhood is geographically variable and ranges from 4% to 26%.^8^ Although the initial symptoms may appear in childhood, BD is rarely diagnosed before the age of 16.^1,7^ The onset of uveitis associated with BD in children also generally occurs in late childhood.^4,7^ Likewise, the incidence of both BD and its ocular manifestations decreases with age.^4^ Disease activity is also observed to decrease in the older age group.^9^ According to a multicenter national database study on the epidemiology of uveitis conducted in Turkey, Behçet’s uveitis (BU) is the leading cause of non-infectious uveitis, accounting for 24.9% of cases. BU is responsible for 9.3% of pediatric uveitis cases and 9.7% of uveitis cases in older adults (>60 years).^10^ In our series, this rate was 16.5% for pediatric uveitis, while BD was not observed among patients diagnosed with uveitis at an advanced age.^11,12^

Although BD is more common among males, there are regional variations in the male/female ratio. In publications from Western Europe, this ratio is quite low and sometimes even higher among females, whereas in publications from Turkey, males outnumber females by at least two fold.^4,11,13,14^ Panuveitis and resulting poor visual prognosis are also more common in males.^1,4,14^

### Etiopathogenesis

Despite better recognition of the disease and numerous studies investigating its underlying causes, there is lingering uncertainty regarding its etiopathogenesis. Disorders of both the innate and adaptive immune systems have been implicated. Environmental factors are believed to play a triggering role in individuals with immunogenetic susceptibility, leading to an increased and abnormal immune response that results in the development of systemic vasculitis.^15,16^ The most well-known genetic link is its association with HLA-B51.^17,18^ Gül et al.^19^ reported that ocular involvement was more common in HLA-B51-positive patients, but there was no relationship with severity of the involvement. It was reported that HLA-A*2601 was significantly more frequent among BU patients in Japan, especially in patients without HLA*B5101, and that HLA-A*2601 was therefore another risk factor for BU in the Japanese.^20^ Other causes implicated in the pathogenesis of the disease include abnormal cellular responses, T-cell-mediated immune responses, abnormal response to bacterial antigens, increased Th1 cytokine production, disorders of the complement system, upregulation of endothelial cell surface molecules, hemodynamics, and coagulation factor abnormalities.^21^ Environmental factors also play an important role. The lower prevalence of the disease among Turks living in Germany is significant evidence of this.^22^ Japan has seen decrements in both the incidence and severity of the disease in recent years. Such changes in a genetically homogeneous country with low immigration rates also suggest the impact of environmental factors. The main reasons for this change in the Japanese population are an increase in atopic/allergic diseases, which are shown to be inversely associated with BD, and a reduction in infectious diseases. Improvement in oral hygiene in particular is the most important factor.^23^ In Turkey, the lower socio-economic status and education level and higher unemployment rate among BD patients compared to patients with ankylosing spondylitis or inflammatory bowel disease further supports the importance of environmental factors.^24^

### Systemic Involvement and Diagnosis of Behçet’s Disease

The underlying pathology is an occlusive, necrotizing vasculitis that can involve arteries and veins of all sizes in all organs and systems. For this reason, the disease is characterized by recurrent inflammatory episodes in affected organs and systems.^25^ The earliest and most common finding is recurrent oral aphthae, which are painful, non-scarring lesions with well-defined borders. In contrast, genital ulcers heal with scarring. Erythema nodosum, papulopustular lesions, acneiform lesions, and increased dermal hypersensitivity reaction to trauma (pathergy) are the most common skin lesions. Other known involvements include superficial thrombophlebitis, deep vein thrombosis, arthritis, epididymitis, and gastrointestinal tract and central nervous system manifestations.^1,4,21^

Diagnosis is based on a constellation of various clinical signs; there is no specific diagnostic test. Positive pathergy test or HLA-B51 positivity alone are not diagnostic findings. There are various recommended diagnostic criteria.^1^ Of these, oral aphthae that recur at least 3 times a year are necessary for the diagnosis of BD according to the criteria established by the International Study Group for Behçet’s Disease. In addition to this, at least two of the following findings are required: Recurrent genital ulcers, cutaneous lesions, uveitis, or a positive pathergy test.^26^ The eye is the most commonly involved organ, with a rate as high as 90% depending on which clinic is performing the study.^27^ Although ocular involvement generally occurs within 2 to 4 years of disease onset, it can be the first sign of the disease in up to 20% of cases.^9,19^ Moreover, ocular involvement is often the complaint that prompts the patient to seek medical care and thus leads to a diagnosis. Therefore, good knowledge of the ocular manifestations of BD is of diagnostic value.

### Ocular Involvement in Behçet’s Disease

Ocular involvement is characterized by bilateral, recurrent, non-granulomatous panuveitis and retinal vasculitis. Isolated anterior uveitis and unilateral involvement are rare.^13^ Posterior segment involvement has been reported in 50-93% of cases. Recurrent episodes of posterior uveitis can result in severe retinal damage and permanent vision loss.^1,13,14,28^ Therefore, recognition of posterior segment involvement also has prognostic value.

BU is characterized by exacerbations and remissions. Sudden, severe attacks followed by spontaneous, gradual remission periods are important findings suggestive of BU.

**Figure 1:**
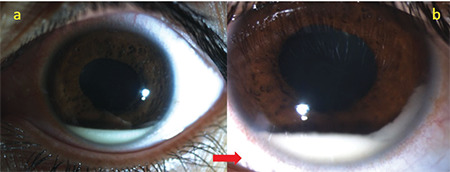
Soft hypopyon (a) that moves freely (b) with head movements (red arrow) is seen in a patient with Behçet’s uveitis. Note that the eye is white despite hypopyon

The prevalence of isolated anterior uveitis is approximately 10%. Anterior chamber reaction is accompanied by dust-like keratic precipitates called endothelial dusting. The eye may appear quiet and white despite a severe anterior chamber reaction and even hypopyon, or there may be anterior segment involvement accompanied by conjunctival hyperemia and ciliary injection. Hypopyon has been reported in 5-30% of cases. However, as hypopyon can regress spontaneously, the actual rate may be higher than reported. The anterior chamber reaction is typically not accompanied by fibrinous exudation, and the inflammatory cells are able to move freely. For this reason, the hypopyon that occurs in BU shifts readily with gravity. The presence of hypopyon is also an indicator of severe posterior segment involvement. These features are essential for distinguishing BU-related hypopyon from ankylosing spondylitis hypopyon, which is sticky with fibrinous reaction and only affects the anterior segment (Figure 1).^1,13,21^

**Figure 2:**
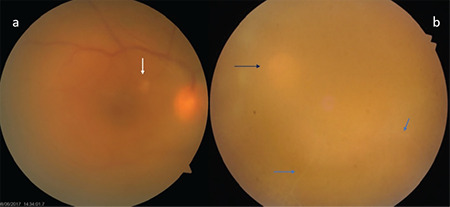
Diffuse vitritis and vitreous haze are observed in two different Behçet’s uveitis patients. In the first patient (a), the optic disc is hyperemic and there is a small retinitis focus (white arrow) at the posterior pole. In the other patient (b), the vitreous haze is very dense and the optic disc (black arrow) and ghost vessels below (blue arrows) are barely discernible

**Figure 3:**
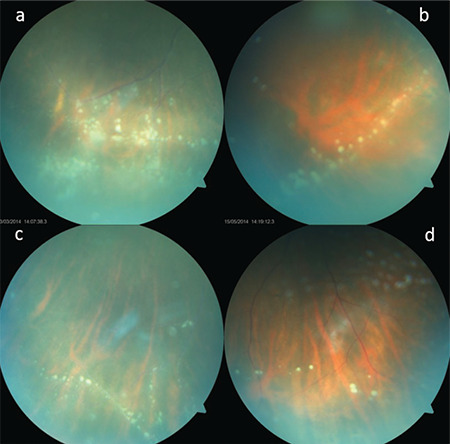
In different patients (a,b,c,d), precipitates (vitreous pearls) are seen in the inferior periphery of the retina, indicating a regressing acute inflammatory episode

The main posterior segment findings are diffuse vitritis with or without vitreous haze, retinal vasculitis, occlusion of major or peripheral retinal veins and less commonly the arterioles, superficial/deep retinal infiltrates, optic disc inflammation, and cystoid macular edema (CME).^14,21^ Diffuse vitritis is an unvarying sign of posterior segment involvement. Vitreous haze is a sign of active inflammation that is most pronounced at the beginning of an attack and gradually diminishes. Sometimes the vitreous haze is so dense that it obscures the posterior segment (Figure 2). As vitreous inflammation regresses, inflammatory precipitates (vitreous pearls) form in a string like a pearl necklace in the inferior peripheral retina (Figure 3).^1,13,21^ This finding is pathognomonic and is an indication that the attack started about 1 week ago and is now regressing. Unlike the snowball opacities in the pars planitis, these are smaller, show an organized arrangement, are mostly located in the inferotemporal retina, and regress spontaneously without scarring.^1^

**Figure 4:**
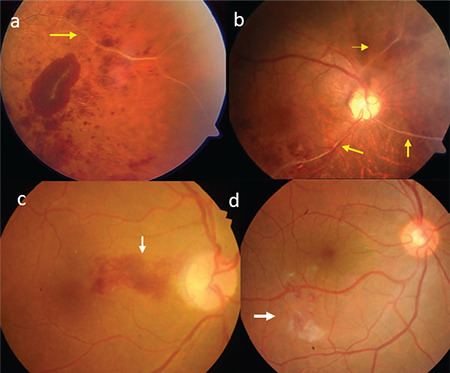
Different patients exhibit perivenous white sheathing (a, b, yellow arrows), hemorrhagic occlusive vasculitis at the posterior pole and associated hemorrhage in the papillomacular bundle (c, white arrow), and hemorrhage and exudates in the inferior macular region (d, white arrow)

**Figure 5:**
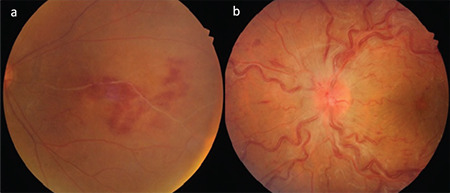
Branch retinal vein occlusion (a) and central retinal vein occlusion (b) due to occlusive retinal vasculitis in two different Behçet’s patients

**Figure 6:**
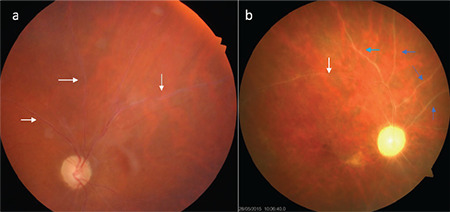
Gliotic sheathing (white arrows) of the retinal vessels and ghost vessels (blue arrows) are observed in two different Behçet’s patients (a, b)

**Figure 7:**
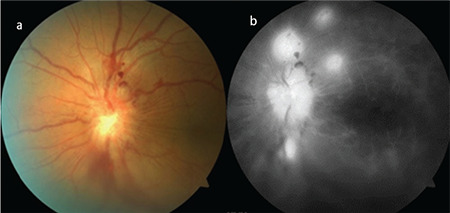
Color fundus photographs (a) and fluorescein angiography images (b) of a Behçet’s patient who developed optic disc neovascularization. There is extensive vascular and capillary leakage

**Figure 8:**
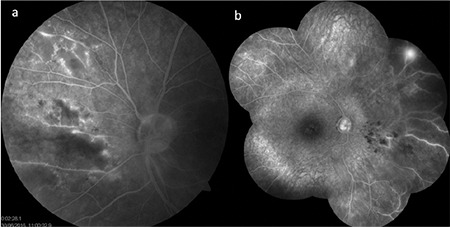
Fluorescein angiography images of two different Behçet’s uveitis patients with occlusive retinal vasculitis. The first (a) shows nasal optic disc ischemia and collateral formation in some areas; a composite image from the other patient (b) shows diffuse ischemia in the nasal periphery and retinal neovascularization

**Figure 9:**
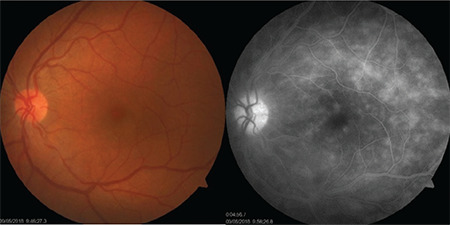
Fluorescein angiography of a Behçet’s patient with no clinically apparent retinal vasculitis on color fundus photograph shows optic disc staining, macular edema, and diffuse capillary and vascular leakage

Retinal vasculitis is another characteristic finding that involves white perivenous sheathing that is often diffuse but can also be segmental.^13,29^ Veins (periphlebitis) are affected more than arteries (periarteriolitis). Periarteriolitis is not seen in isolation; it is always accompanied by periphlebitis. Capillaritis is also a common finding that leads to diffuse capillary leakage and is best observed with fluorescein angiography (FA). The characteristic feature of periphlebitis associated with BU is that it is occlusive, leaky, and recurrent. It can affect vessels in any location and of every size. Occlusive vasculitis may lead to retinal hemorrhage and exudations, and even the formation of branch retinal vein occlusion or more rarely, central retinal vein occlusion (Figures 4, 5). After the active inflammation has subsided, findings including armor-like gliotic sheathing of the internal vascular wall, ghost vessels (Figure 6), and retinal ischemia demonstrated by FA may be observed.^1^ Retinal neovascularization (NVE) and less commonly neovascularization of the disc (NVD) may also occur as a complication of retinal ischemia (Figures 7, 8).^1^ The underlying cause of NVD is not ischemia but uncontrolled inflammation, and its treatment should be targeted accordingly. Tutkun et al.^30^ reported that ischemia was present in only 13% of cases who developed NVD due to BU. Sometimes retinal vasculitis is not observed clinically, but manifests as subclinical chronic vasculitis demonstrated by FA. Optic disc staining and retinal capillary leakage observed on FA during a clinically calm period between attacks are key signs of persistent subclinical inflammation (Figure 9).^28,31^

**Figure 10:**
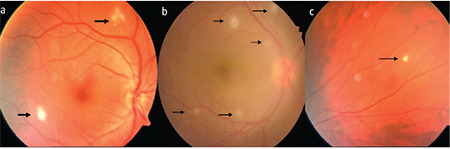
Superficial retinal infiltrates (arrows) are observed in different patients (a, b, c). Even a single one (c) is regarded as posterior segment involvement

**Figure 11:**
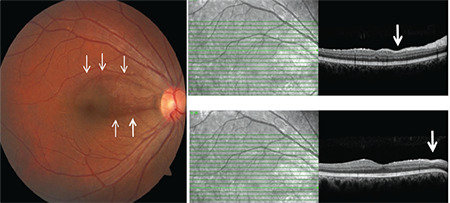
Color fundus photograph of a Behçet’s uveitis patient shows a wedgeshaped localized retinal nerve fiber layer loss (arrows) in the superior macula and the papillomacular bundle and thinning (arrow) on SD-OCT sections corresponding to the area of loss

Superficial and deep retinal infiltrates are the most common findings of posterior segment involvement of BU. Superficial infiltrates heal within a few days without scarring. Even without accompanying retinal vasculitis, the presence of even one of these infiltrates is considered an indicator of posterior segment involvement (Figure 10). Deep retinal infiltrates take longer to heal and may leave a scar. The wedge-shaped defect in the retinal nerve fiber layer (RNFL) left as retinal infiltrates in the posterior pole regress, and visual field loss and RNFL thinning on optical coherence tomography (OCT) in the region corresponding to this defect have been identified as characteristic findings for patients with BU (Figure 11).^32^

**Figure 12:**
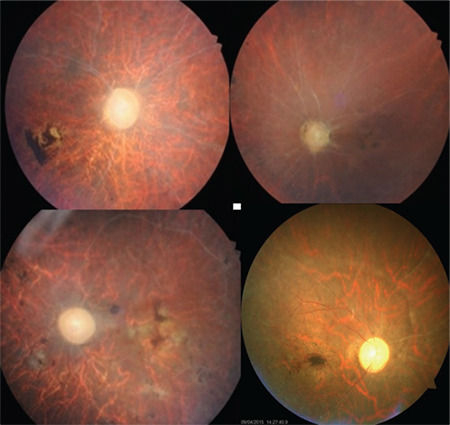
The appearance of the fundus in different patients with end-stage disease. Optic atrophy, macular scarring, retinal atrophy, ghost vessels, and areas of retinal pigmentation can be seen

**Figure 13:**
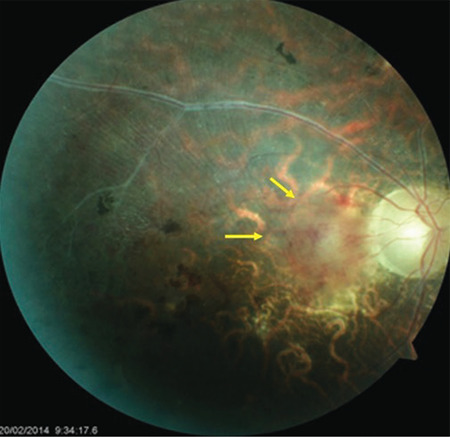
Active vasculitis in the papillomacular bundle (yellow arrows) is observed in an end-stage eye with retinal and macular atrophy, gliotic sheathing, pigmentation, and optic atrophy

The main anterior segment complications of BU include cataract, posterior synechiae, and glaucoma. Posterior segment complications are more numerous and many have the potential to cause permanent vision loss. These include macular edema, optic atrophy, retinal atrophy, macular scarring, epiretinal membrane, retinal detachment, retinal tears, NVE, NVD, macular holes, and even phthisis bulbi. Macular complications and optic atrophy are the leading causes of permanent vision loss.^1,10,11^ The clinical picture in the most advanced stage of the disease (end-stage, terminal disease) is characterized by optic atrophy, ghost vessels, varying degrees of pigmentation, diffuse retinal atrophy, gliosis, macular scarring, and a transparent vitreous (Figure 12).^1^ This clinical presentation can sometimes be confused with retinitis pigmentosa. Even patients with end-stage disease can sometimes have new activations (Figure 13).

### Imaging in Behçet’s Uveitis

**Figure 14:**
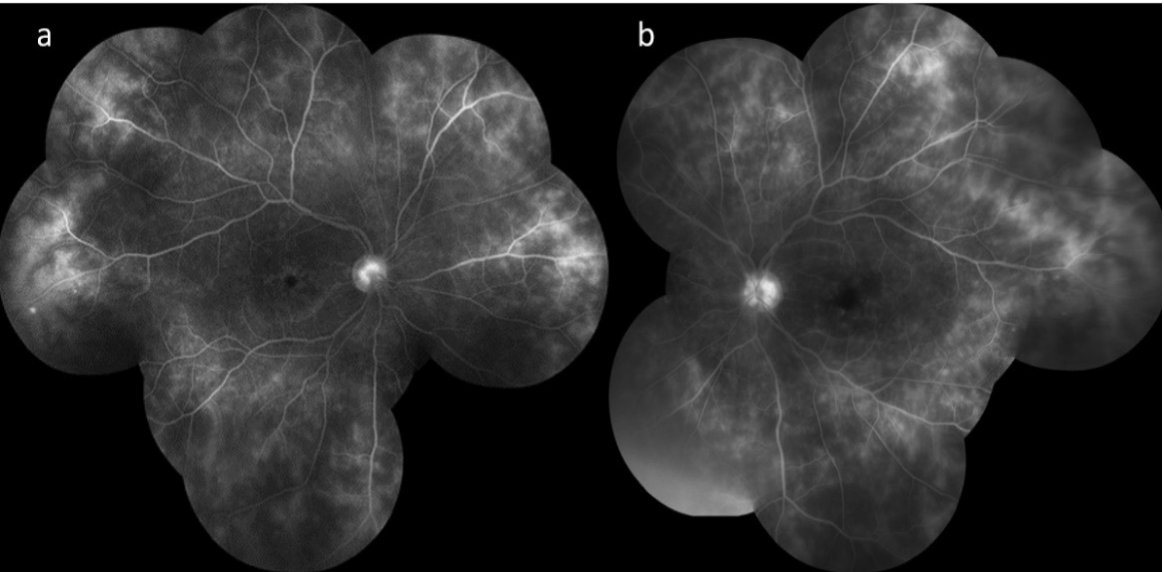
Bilateral (a,b) optic disc staining, cystoid macular edema, vascular leakage, and fern-shaped capillary leakage are noted on fluorescein angiography in a Behçet’s patient

Color fundus photography is a method we often use to visualize and monitor BU lesions. Demonstrating vitreous haze, retinal infiltrates, and the spontaneous regression of the vitreous precipitates observed in the inferior peripheral retina is particularly helpful in distinguishing from other possible causes.^31^ Despite all of the advances in imaging methods, FA is still the gold standard for detection and monitoring of the occlusive and leaky vasculitis caused by BU.^31,33^ The most important FA findings of active BU include dilation and increased tortuosity of the retinal veins, vascular leakage, and leakage from the optic disc, macular, and retinal capillaries. Fern-like capillary leakage is the most characteristic FA finding of BU as well as an important indicator of activity (Figure 14). This finding shows that inflammation is active even if the uveitis appears calm clinically and indicates that the current treatment is inadequate. The extent and occlusivity of retinal vascular involvement, capillary non-perfusion areas, collateral vascular formations, and neovascularization are best visualized with FA.^31,33^ The need for laser photocoagulation (LPC) is also determined based on FA findings. As mentioned above, most NVD exhibit diffuse capillary leakage rather than ischemia as an indicator of persistent inflammation. Therefore, the treatment is not LPC, but rather strengthening the anti-inflammatory therapy.^30^ FA findings also have prognostic value. In various studies, FA findings such as NVD, macular window defect, macular ischemia, macular leakage, posterior and diffuse retinal vasculitis, excessive retinal vascular leakage, optic disc hyperfluorescence, peripheral capillary non-perfusion, CME, and arterial narrowing have been associated with poor visual prognosis.^34,35,36,37^ For this reason, the focus shifted to angiographic classification and staging of Behçet’s retinal vasculitis and monitoring activation accordingly.^35,38,39^ Keino et al.^40,41^ reported that after 1 year of infliximab (IFX) therapy, there were decreases in both ocular inflammatory episodes and retinal vascular leakage and disc leakage. When the same authors evaluated the effect of IFX over a 4-year period, they demonstrated that mean retinal vascular and disc leakage scores decreased further after each year of treatment.^41^

**Figure 15:**
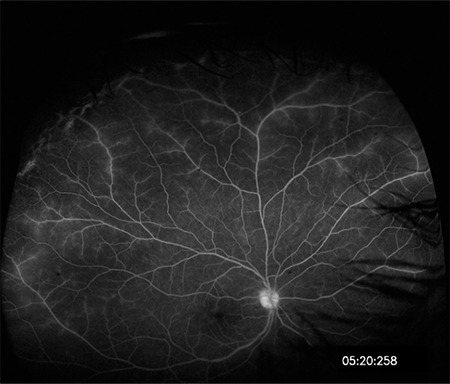
Fluorescein angiography with ultra-wide-field imaging shows vascular leakage in the superior and temporal periphery in addition to the optic disc and macular leakage. Shadowing caused by the lashes is present in the inferior and nasal regions

With conventional fundus cameras, images limited to 30°-60° can be obtained and the entire retina cannot be visualized simultaneously. The ultra-wide-field imaging system (OptosPLC, Scotland, UK) makes it possible to obtain fundus photographs and autofluorescence and angiography images of a 200° field.^31^ Studies comparing clinical examination with conventional and ultra-wide-field imaging have shown that wide-field imaging contributes significantly both to detection of disease activity and treatment decision-making.^42,43,44^ A recent study by Jones et al.^43^ compared standard 7-zone FA with an ultra-wide-field imaging system in a series of 106 cases of retinal vasculitis. It was reported that 43.4% of lesions detected with wide-field imaging could not be visualized with standard FA and that a large portion of treatment modifications were made based on the lesions detected by wide-field imaging.^43^ Peripheral retinal vascular involvement due to Behçet’s retinal vasculitis and the associated leakage, ischemia, and neovascularization are quite difficult to demonstrate with standard FA. Therefore, visualizing the peripheral retina with ultra-wide-field imaging contributes significantly to the diagnosis, monitoring, and treatment of Behçet’s vasculitis (Figure 15).^31^ In fact, the use of wide-field imaging in 20 Behçet’s patients with active retinal vasculitis revealed additional findings requiring treatment changes in 80% of the patients. It is notable that peripheral retinal non-perfusion was observed in 66.7% of the eyes. Based on wide-field imaging findings, immunomodulatory therapy was modified in 65% of the patients and LPC was performed on 10.5% of eyes.^44^

As BD is a systemic vasculitis, involvement of the choroidal vasculature is also expected. The method that best shows the choroidal vascular structure is indocyanine green angiography (ICGA). The ICGA findings seen in BU have been demonstrated in various studies.^45,46,47^ These findings are not specific to BU, but include filling delay/defect of the choriocapillaris, hyperfluorescence of stromal vessels, staining of the choroidal vascular walls, hyperfluorescent spots, hyperfluorescent plaques, and hyperfluorescence in the optic disc and diffuse hyperfluorescence in the choroid in the middle or late phase of ICGA.^45,46,47^ It has been shown that these findings are not significantly associated with systemic findings of BD. Likewise, it is believed that there is no remarkable relationship between FA and ICGA findings, that ICGA does not provide additional information regarding disease activity and treatment monitoring, and therefore is unnecessary in the routine follow-up of BU. ICGA is used more for differential diagnosis than diagnosis.^31^

There are not many studies regarding the use of fundus autofluorescence (FAF) imaging in BU. In a study conducted with ultra-wide-field FAF, it was reported that active retinal vasculitis may lead to retinal pigment epithelium (RPE) changes in the peripheral retina, with 82.3% of patients showing such changes.^44^ Our view is that FAF does not make an additional contribution in the follow-up of BU.^31^

**Figure 16:**
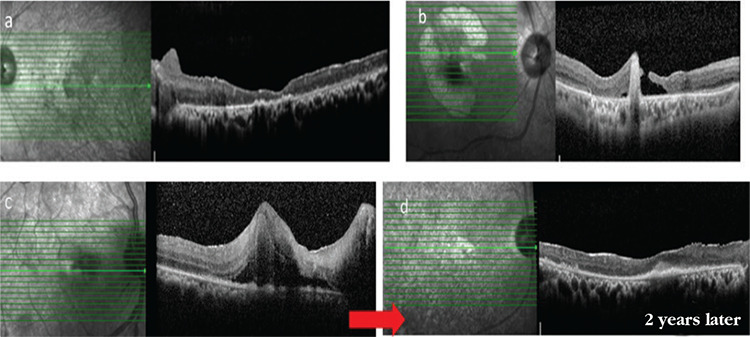
Macular atrophy in a patient with advanced Behçet’s uveitis (a), macular atrophy and hole in another patient (b), a patient who presented with active retinitis involving the macula and associated macular edema (c), and the same patient 2 years later, exhibiting disorganization and atrophy of the retinal layers and subfoveal fibrosis (d)

OCT is a method that non-invasively shows posterior pole lesions and macular complications and is frequently used in the follow-up of BU. Although FA is the best method for evaluating the general uveitis activity, OCT is superior in demonstrating macular edema and identifying its pattern. Only OCT can show whether the fluid is diffuse, cystoid, or located subretinally.^48^ With the introduction of OCT, it has been shown that BU can cause not only CME, but also serous macular detachment.^49^ Vitreoretinal interface disorders are also best demonstrated by OCT. The incidence of interface disorders was shown to be associated with uveitis duration.^50^ Complications such as epiretinal membrane, vitreomacular adhesion, vitreomacular traction, lamellar or full-thickness macular holes, macular atrophy, and macular scarring are best visualized with OCT. Spectral domain (SD)-OCT also enables evaluation of the outer retinal layers (Figure 16). The integrity of the ellipsoid zone (inner segment [IS]/outer segment [OS] band) and interdigitation zone is closely associated with visual function and prognosis in eyes with uveitic macular edema. The foveal thinning and ellipsoid zone irregularity shown on OCT reflect irreversible damage to the macula caused by BU and are an indicator of poor visual prognosis.^31^ In a recent study, Kang et al.^51^ examined whether central macular thickness (CMT) and macular volume values measured with SD-OCT were associated with the uveitis severity in BU patients without macular edema. Mean CMT and macular volume were significantly higher in patients with posterior involvement and decreased with treatment. They reported that OCT is a useful adjunctive method in BU follow-up, especially for identifying posterior segment involvement, that it would reduce unnecessary FA imaging, and that it is also useful for treatment monitoring. However, as the authors also acknowledged, OCT cannot replace FA in the follow-up of BU because it does not demonstrate the current state of the retinal vasculature. In addition, since macular thickening may occur independent of disease activity in eyes with permanent vascular damage, follow-up with OCT alone is misleading in chronic cases.^51^

**Figure 17:**
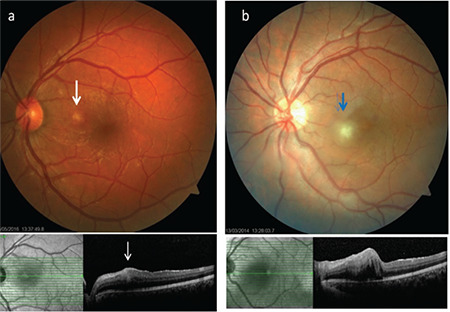
The superficial retinal infiltrates (a, white arrow; b, blue arrow) associated with Behçet’s uveitis led to focal retinal thickening and blurring and increased hyperreflectivity in the inner retinal layers in particular, while the contour of the retinal pigment epithelium was not disrupted

**Figure 18:**
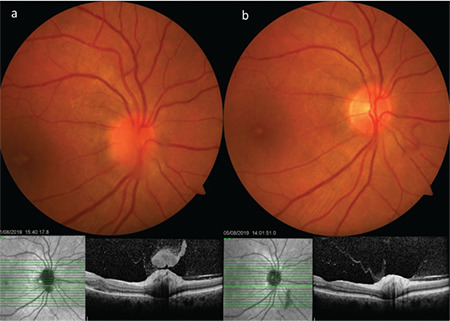
A patient with Behçet’s neuroretinitis exhibits optic disc infiltration accompanied by vitreous inflammation that looks like a hat over the disc on SDOCT, as well as subfoveal fluid and cystic edema (a). Four days after intravenous methylprednisolone therapy, substantial regression of the optic disc infiltration, overlying vitreous inflammation, and macular edema are observed (b)

Nevertheless, the use of OCT has improved our understanding of the structure of BU lesions and the damage they cause. Transient superficial white infiltrates are the most common lesions seen in BU exacerbations. SD-OCT sections obtained from over these retinal infiltrates show focal retinal thickening, blurring of the inner retinal layers, as well as increased hyperreflectivity and optical shadowing (Figure 17). Unlike in retinochoroiditis, there is no choroidal thickening below the retinal infiltrates and the RPE contour is not disrupted. These infiltrates disappear quickly without leaving a clinically apparent scar. However, SD-OCT sections have shown that internal retinal atrophy develops in this region and that the superficial retinal infiltrates at the posterior pole leave localized non-glaucomatous defects in the RNFL (Figure 11).^31,32,33,52^ Papillomacular or arcuate RNFL defects, which can be demonstrated very well with SD-OCT, also lead to localized visual field defects.^32,52^ These localized RNFL defects are a diagnostic finding indicative of posterior pole involvement in early BU but cannot be observed in end-stage disease due to diffuse retinal and optic atrophy.^31^ In Behçet’s neuroretinitis, the localized vitreous inflammation that appears like a hat over the optic disc infiltration and its regression can also be observed non-invasively on SD-OCT (Figure 18).^31^

Numerous enhanced depth imaging (EDI)-OCT studies have been conducted in BU patients and they have yielded conflicting results. One study demonstrated that subfoveal choroidal thickness is greater during the acute stage compared to the remission period and is associated with clinical inflammation scores, while another study showed that thickness was not related to uveitis severity or duration.^31^ There are even studies indicating that the choroid is thinner in patients with active posterior uveitis or that choroidal thickness does not differ between patients experiencing acute episodes and those who are in remission. It has been suggested these differences in results stem from the inhomogeneity of the patient populations, differences in activity and remission criteria, and varying disease durations. The fact that choroidal thickness shows individual variations also contributes to these conflicting results. For this reason, automated central foveal thickness measurement by OCT is still a more useful method for evaluating the inflammatory activity of BU.^31^ A fairly recent study by Onal et al.^53^ quantitatively evaluated choroidal structural changes in patients with active BU. It was shown that there was enlargement of the choroidal stroma in the patient group compared to the control group, but that this did not lead to an increase in choroidal thickness or make a difference in terms of subfoveal choroidal thickness. In contrast, the authors stated that central foveal thickness measurement is a useful and non-invasive method for evaluating inflammatory activity in early BU. In their study, central foveal thickness was shown to be significantly associated with visual acuity, BU ocular episode score, and total FA and ICGA scores.^53^ The studies of both Onal et al.^53^ and Kang et al.^51^ show that CMT measurement is an easily applicable method for assessing activity in patients with early BU, who do not have macular edema or macular and optic atrophy.

Optical coherence tomography angiography (OCTA) is a newer imaging method that demonstrates retinal and choroidal vascular morphology. There are few studies on its use in cases of BU.^54,55,56^ In their first study, Khairallah et al.^54^ reported that the foveal avascular zone was larger and capillary vessel density was lower in the BU group compared to the control group, and that OCTA was superior to FA in demonstrating perifoveal microvascular changes. It was also shown that impaired capillary perfusion and capillary network anomalies were more pronounced in the deep capillary plexus compared to the superficial capillary plexus.^54^ Subsequent studies have also supported these findings.^55,56^

### Treatment of Behçet’s Uveitis

The path to preventing recurrent episodes of uveitis and the resulting ocular complications, and thus improving visual prognosis, lies in effective treatment. There are several goals in the treatment of BU. Quickly suppressing acute episodes to prevent tissue damage and restore potential vision is the primary goal, but is not sufficient. Additional goals include suppressing chronic subclinical inflammation to prevent possible complications, preventing recurrences, and maintaining achieved remission, thereby preserving vision.^57^

At present, corticosteroid (CS) monotherapy has no place in the treatment of BU, and posterior segment involvement definitely requires the use of immunosuppressive or immunomodulating agents.^58^ However, CSs are still used for the treatment of acute inflammatory episodes. When a rapid response is desired, the most commonly used treatment protocol consists of 1 g/day intravenous (IV) pulse methylprednisolone for 3 days, followed by high-dose oral prednisone (1 mg/kg/day) which is tapered gradually and reduced to the maintenance dose (≤7.5 mg) after active inflammation has been suppressed.^21,59^ Starting with a high oral dose (1-1.5 mg/kg) is another option. Immunosuppressive agent(s) should be started simultaneously and used in conjunction with CSs until they take effect. Periocular or intravitreal CSs can be used as an adjunctive therapy in cases where systemic CSs cannot be used or an adequate response is not achieved, and especially in patients with a unilateral panuveitis episode and/or refractory CME.^60,61^ It should not be forgotten that BD is a systemic disease and should therefore be treated systemically. When treatment must be intensified or switching to a biologic agent is necessary, CS injections should be kept in mind as a convenient and time-saving adjunctive therapy.^57,61^ Markomichelakis et al.^62^ reported that a single-dose IFX infusion was faster acting than IV or intravitreal CS in the suppression of acute episodes. Therefore, it is a good option for this purpose, but its use as a first-line treatment agent is not currently feasible in Turkey.

In cases of isolated anterior uveitis, treatment with potent CS drops at high initial frequency and tapered to discontinue after 6-8 weeks and mydriatic and/or cycloplegic agents started at 2-3 times a day and discontinued at 2-3 weeks is sufficient.^21^

For posterior segment involvement, the most commonly used conventional treatment agents are antimetabolites (azathioprine [AZA], mycophenolate mofetil, methotrexate), T-cell inhibitors (cyclosporine-A [CSA], tacrolimus), and alkylating agents (cyclophosphamide, chlorambucil). Of these, only AZA and CSA were shown to be effective in randomized controlled trials.^63,64,65,66^ These trials have also been supported by many clinical studies.^67,68,69,70^ Despite the introduction of many new molecules, AZA and CSA are still the most commonly used agents, either alone or in combination. They are also known to be more effective when used in combination.^71^ Complete blood count and liver function tests should be followed for AZA, while complete blood count, kidney function, blood pressure, and development of gingival hyperplasia and hirsutism should be followed when treating with CSA. Another important point to consider about CSA is that it should not be used by patients with neurological involvement.^72^ Although no study has investigated the use of mycophenolate mofetil to treat BU specifically, there are studies showing that this drug is effective in uveitis patients, which also included those with BU.^73^ Although alkylating agents are still used for some extraocular involvements of BD (acute deep vein thrombosis, arterial involvement), they are not preferred in cases of ocular involvement due to serious adverse effects, such as development of malignancy, and the current availability of biologic agents. Although colchicine is effective for the mucocutaneous symptoms of BD, its efficacy against ocular involvement has not been demonstrated.^21,74^

More potent and faster acting agents are needed for patients who are non-responsive to conventional treatment, those who have frequent recurrences, and those who present with severe posterior segment involvement and vision loss. Currently, biologic agents are used for this purpose. In 2018, EULAR (European League Against Rheumatism) updated its 2008 recommendations for the treatment of BD.^74,75^ The updated EULAR recommendations also broadened the areas of use of biologic agents in the treatment of BU. While they formerly recommended starting BU patients with posterior segment involvement on AZA and CS therapy and adding CSA or IFX or switching to interferon-alpha (IFN-a) for non-responders, they now recommend initiating AZA, CSA, INF-alpha, or monoclonal anti-tumor necrosis factor (TNF) therapy for the treatment of posterior segment involvement. It is emphasized once more that CSs should not be used alone, but rather in combination with AZA or other immunosuppressants. It is also stressed that high-dose CSs, IFX, or IFN-a-2a should be used to treat patients presenting with first-time or recurrent vision-threatening acute uveitis. In other words, the use of biologic agents as first-line therapy is recommended in selected patients. Intravitreal CS injection is recommended as an adjunct to systemic treatment in patients with unilateral episodes.^75^ Expert recommendations for the use of anti-TNF agents to treat ocular inflammatory diseases published by Levy-Clarke et al.^76^ also recommended IFX and adalimumab (ADA) as first-line treatment for BU only, and second-line therapy for all other causes.

The human-mouse chimeric monoclonal antibody IFX and the completely human protein-based ADA are the anti-TNF agents most commonly used in the treatment of BU. Published studies show that both agents effectively treat refractory BU through the rapid and potent suppression of ocular inflammation. They are known to reduce both the frequency and severity of uveitis episodes. Anti-TNFs reduce the optic disc and vascular leakage observed on FA, enable substantial CS cessation, and are generally well tolerated.^40,41,77,78,79,80,81,82,83^ When conventional therapy and IFX were compared with respect to the treatment of Behçet’s retinal vasculitis, it was shown that with IFX, the mean remission period was longer (17 months vs 5 months), the average number of episodes in 24 months was lower (1.2 vs 6.3), visual outcomes were better (the prevalence of optic atrophy was 30% with IFX and 60% with conventional therapy), and there were fewer ocular and systemic complications.^84^

If IFX and ADA were compared, the conclusions would be that both effectively suppress uveitis, that IFX has a fast-acting and potent anti-inflammatory effect equivalent to that of IV pulse methylprednisolone but should be combined with an antimetabolite due to its high immunogenicity (autoantibody formation, loss of effect, infusion reaction), whereas ADA is more effective at inducing sustained remission and is safer and more appropriate as monotherapy due to its lower risk of immunogenicity. Another difference is how they are used. IFX is administered IV in hospital conditions, while ADA is administered subcutaneously.^76,85,86^ The first study to compare these 2 anti-TNF agents in BU patients resistant to conventional therapy was published in 2019 and confirmed that both agents were effective.^87^ However, it was also reported that after 1 year of treatment, patients using ADA had better outcomes, and in particular showed significantly greater improvement in visual acuity and treatment continuation rate.^87^ Another fact that should be regarded as being in favor of ADA is that it is the only biologic agent tested in randomized controlled trials and approved for the treatment of non-infectious uveitis.^88,89^

Another biologic agent that is often used to treat Behçet’s uveitis and whose efficacy has been demonstrated in many studies is IFN-a-2a. It provides complete or partial improvement of inflammation at rates of up to 98% and improves or stabilizes vision when used to treat BU. It takes effect within 2 to 4 weeks. It has been reported that due to its antiangiogenic activity, it also leads to reperfusion of occluded vessels and regression of neovascularization.^30,90,91,92,93,94,95,96,97,98^ There is no standard usage. Some recommend starting at a high dose and then tapering after a response is achieved, while others prefer to start with a low dose and increase the dose according to the response achieved. Due to its potential myelosuppressive effect, it should not be used together with other immunosuppressants. The main adverse effects include the influenza-like symptoms experienced by nearly all patients, especially at the beginning of treatment, as well as alopecia, elevated liver enzymes, thyroiditis, autoantibody formation, weight loss, and depression.^21,97^ The most important advantage of IFN-a-2a is that it can provide long-lasting remission that persists even after treatment is discontinued, and that the same effectiveness can be attained if treatment must be reinitiated.^95,96^

When Ozgüler et al.^97^ compared studies in which IFN-a and IFX were used to treat BU, they reported that IFX took effect more rapidly (24 hours) and improved visual acuity in more cases (76% vs 46%), but that rates of sustained remission (71% vs 44%) and CS cessation (66% vs 33%) were higher with IFN. Rates of complete or partial remission and drug discontinuation due to adverse effects were similar.^97^ Yalçindağ and Köse^98^ conducted the only study comparing IFN-a and IFX in BU patients resistant to conventional therapy and reported that there was no difference between the agents in terms of anti-inflammatory activity or visual acuity improvement, while there were more adverse effects with IFN.

In cases where an adequate response is not achieved even with biologic agents, instead of using high-dose CS, the current biologic agent should be increased in dose and/or frequency or treatment should be switched to an alternative biologic. Tocilizumab, an anti-interleukin-6 (IL-6) receptor antibody, was used to treat 5 BU patients resistant to IFN-a and anti-TNF-a therapy, and all of the patients showed both clinical and angiographic improvement as well as a significant reduction in CMT.^99^ Another molecule reported to be successful in treating resistant patients is golimumab, which is also an anti-TNF-a agent. It was shown to induce rapid regression of retinal vasculitis and reduce ocular episodes in 5 patients resistant to conventional and other biologic therapies.^100^ Another alternative may be the use of the IL-1 inhibitors anakinra and canakinumab. Fabiani et al.^101^ reported that Behçet’s patients with uveitis of long duration in particular responded better to IL-1 inhibitors. Studies on pegylated IFN-a, secukinumab, daclizumab, gevokizumab, and rituximab showed they were not sufficiently effective.^97^

**We can summarize our current approach to the treatment of Behçet’s uveitis as follows: **Every patient with posterior segment involvement is started on conventional therapy consisting of AZA ± CSA. If the patient presents during an acute episode, we also add systemic CS, aiming to taper the dose slowly and discontinue within 3 months. As second-line treatment we use biologics, of which IFN-a is our first choice. In patients who are non-responsive to this treatment, we switch to anti-TNF agents. If there is severe, vision-threatening involvement at the time of admission, we try to switch to biologic agents without wasting too much time with conventional agents. Periocular or intravitreal CS injections are used as adjunctive therapy in patients with severe involvement whose treatment we plan to change and especially in patients with unilateral exacerbations or a condition that precludes systemic CS use.

Before initiating a systemic conventional or biologic therapy, all patients should be evaluated in terms of complete blood count, liver and kidney function tests, systemic comorbidities, infectious diseases like hepatitis and tuberculosis (TB), history of malignancy, mental state, pregnancy/breastfeeding, and immunization history. Patients should be screened for risk of TB and demyelinating disease before using an anti-TNF agent.^102^ Among the rheumatoid diseases, BD poses the highest risk for TB. Anti-TNF agents increase this risk. The risk with IFX is reported to be 2 fold higher than with ADA.^103^ For patients with an induration >5 mm on tuberculin skin test and/or positive QuantiFERON test, it is recommended to start isoniazid prophylaxis 1 month before anti-TNF therapy and continue for 9 months. In Turkey, the Ministry of Health issued a guide for the management of TB in patients using anti-TNF.^104^ Anti-TNFs should not be used by patients with a history of demyelinating disease, and those with a family history should be informed of the risk.^85^

As mentioned above, FA is the gold standard in treatment monitoring. However, the laser flare meter, which objectively assesses the presence of inflammation by measuring the amount of protein in the anterior chamber, is also an important tool in the follow-up of BU. Tuğal-Tutkun et al.^105^ demonstrated that there was a significant relationship between laser flare meter measurements and anterior chamber cells, vitreous haze score, and fundus lesions in BU patients. It was also shown in patients in clinical remission that anterior chamber flare score and FA leakage score were significantly associated and that those with flare measurements over 6 photons/ms were more likely to have recurrence. Therefore, laser flare meter measurements can be used in patient follow-up as an adjunctive method that demonstrates the presence of chronic refractory vasculitis and reduces the need for FA.

**Figure 19:**
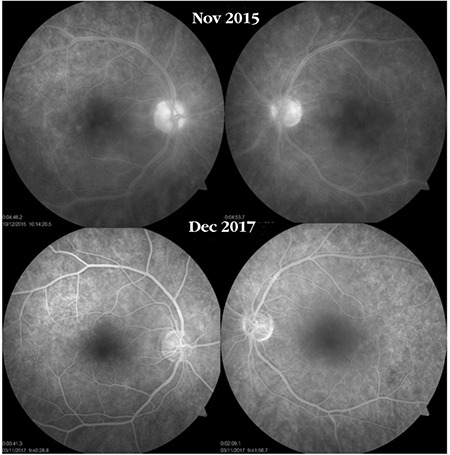
A Behçet’s uveitis patient with bilateral optic disc staining, macular edema, and vascular and capillary leakage shows marked improvement approximately 2 years after interferon-alpha therapy

There is still no definitive answer to the question of when to discontinue treatment. Clinical improvement of uveitis does not mean that the disease is inactive. Treatment effectiveness should be evaluated based on clinical symptoms together with FA findings (Figure 19). There must be no signs of retinal vascular and capillary leakage on FA to say that complete remission has been achieved. Generally, we use the immunosuppressive/biologic agent for at least 2 years, and if clinical and angiographic remission are observed we continue treatment while reducing the drug dose and/or lengthening the infusion/injection intervals for another year with periodic FA examination, and finally discontinue treatment. Patients should be followed closely even treatment discontinuation.

### Prognosis of Behçet’s Uveitis

Despite reports that the course of BD has become milder in recent years due to advances in treatment, changing environmental factors, and increased awareness regarding the disease, it still has a high potential for blindness.^106^ The most important determinant of visual prognosis is cumulative damage caused by recurrent episodes involving the posterior segment. The main factor in improving prognosis is developments in therapeutic agents and our understanding of treatment. The introduction of CSA in the 1990s and of biologic agents in the 2000s, the abandonment of CS monotherapy, earlier initiation of immunomodulatory therapy, and the use of combined treatment regimens have improved visual prognosis.^13,107,108,109^

## Conclusion

BU is the leading cause of non-infectious uveitis in Turkey. It is characterized by recurrent episodes of non-granulomatous panuveitis and occlusive retinal vasculitis. As it is more common among young adults and is potentially blinding, early diagnosis and potent treatment are crucial.
